# Hydrogeochemical and isotopic investigations of groundwater in the reclaimed desert located between EL Nasr canal and Mariut Tableland, NW Coast, Egypt

**DOI:** 10.1038/s41598-024-70852-2

**Published:** 2024-09-11

**Authors:** Ahmed K. Alezabawy, Mustafa Eissa, Zenhom El-Said Salem

**Affiliations:** 1 Geology Department, Faculty of Science, Helwan University, Cairo, Egypt; 2 Division of Water Resources and Arid Lands, Hydrogeochemistry Department, Desert Research Center, Cairo, Egypt; 3 Geology Department, Faculty of Science, Tanta University, Tanta, 31527 Egypt

**Keywords:** Groundwater salinization, Hydrochemistry, Stable isotope, Desert reclamation, Influencing factors, El-Nasr canal irrigation area, Egypt, Environmental chemistry, Environmental impact, Hydrology

## Abstract

A complete understanding of groundwater dynamics and its interaction with surface water under the impact of agricultural activities is vital for local agriculture, ecology, and residents of dry regions, which is not commonly recognized in arid areas. This research outlines the geochemical characteristics, recharge sources, and potential factors impacting groundwater quality in a new land reclamation located in the small basin of Abu Mina, which is part of the Western Nile Delta region.1 Thirty-one groundwater samples and two surface water samples were collected in 2021 to represent the Pleistocene aquifer and were subjected to multivariate statistical, hydrochemical, and stable isotope analyses. Data analysis demonstrates that Na^+^ > Ca^2+^ > Mg^2+^ > K^+^ and SO_4_^2–^ > Cl^–^ > HCO_3_^–^ > NO_3_^–^ are the predominant cations and anions, respectively. Groundwater salinity ranged from 465.60 to 6455.18 mg/l, with slightly alkaline. Most of the water samples fall into one of three types of facies: Ca–Cl, Na–Cl, and Mixed Ca–Mg–Cl, in decreasing order. The meteoric genesis index (r2) indicates that deep meteoric water percolation dominates the Pleistocene aquifer. The aquiline diagrams, correlation matrix, and different ionic ratios indicate that evaporation, reverse ion exchange reactions, and the dissolution of carbonate and silicate minerals are the main processes governing groundwater chemistry. Factor analysis (FA) indicated that three factors explain groundwater hydrochemistry, accounting for 71.98% of the total variance. According to the rotating components matrix (F1–F3), the chemistry of the Quaternary aquifer is principally affected by evaporation, ion exchange reactions, and anthropogenic influences. Additionally, salinity increases due to the return flow of irrigation activities and mixing between old and recent water. The stable isotopes (δ^18O^ and δ^2H^) indicate that the Quaternary aquifer receives groundwater recharge through the return flow of excess irrigation and canal seepage. Under desert reclamation conditions, groundwater salinization processes should be given special consideration. All groundwater samples are appropriate for agricultural irrigation based on the Sodium Adsorption Ratio (SAR), Permeability Index (PI), Percent Sodium (%Na), and Residual Sodium Carbonate (RSC).

## Introduction

Groundwater is an essential water resource in arid environments. It is crucial for providing drinking water and supporting irrigated agriculture. Population growth and economic development are major challenges for Egypt’s water resources sector. The Egyptian government has targeted the research region for reclamation initiatives, necessitating the assessment of water resources’ viability and their capacity to support future growth^1^.

Egypt’s groundwater resources are influenced by a variety of factors, including climate change, altering rainfall amounts, patterns, and frequencies, associated drought events, changes in temperature cyclicalities, and changes in land cover and land use^2,3^. Numerous factors can cause groundwater salinity, including seawater intrusion, ion exchange reactions, the dissolution of secondary minerals, the flow of saline water from neighboring aquifers, the return flow contributions of surface water systems, evapotranspiration, anthropogenic input, climate change, and land use. Most of these sources are typically integrated^4–8^.

The northwestern region of the Egyptian Delta has undergone significant changes in recent decades as a result of extensive agricultural projects and land reclamation. This has resulted in a range of ecological and environmental issues, including the fall of groundwater levels, waterlogging of the soil and the deterioration of its quality, as well as challenges to regional sustainable development^9^. Irrigation water quality is evaluated by looking at its chemistry and how it is used. Due to the presence of soluble salts in all bodies of water, whether on the surface or underground, the concentration of the soil solution increases when irrigation occurs^10^. Although prior studies conducted, there is still a significant lack of comprehensive evaluation of water quality in the reclaimed areas of the Abu Mina depression, which is located on the northwest coast of Egypt.

The research area is predominantly agricultural, industrial, and urban, which significantly impacts groundwater conditions. Groundwater levels and quality have changed greatly in the Western Nile Delta due to ancient agricultural practices^1,11^. Understanding the factors that lead to groundwater salinity and the impact of irrigation on salinity is crucial for the long-term sustainability of irrigation systems^12^. The effects of prolonged irrigation on groundwater systems depend on various factors, including drainage conditions, hydrogeochemical context, and irrigation water chemistry^13^.

Numerous methodological studies have examined the main variables affecting groundwater chemistry in the Northern Nile Delta, Egypt. Multivariate statistical analysis is a proven method for grouping water samples and detecting significant factors controlling water quality. Factor analysis is a suitable technique for identifying correlations between data relevant to fundamental, indirectly visible characteristics^7,14^. Understanding the water cycle interaction is essential for creating and managing water resources in arid regions. The interactions between rainwater, surface water, and groundwater are crucial for this understanding^15^.

Stable isotopes of hydrogen and oxygen (δ^2H^ and δ^18O^) in groundwater are frequently used in hydrological and hydrogeological studies as natural tracers^16^. In general, the stable isotopic compositions of the groundwater body remain intact until it becomes mixed with other water that has altered stable isotopic signatures ^17^. They are effective to figure out the primary flow processes and groundwater origin, analyzing the patterns of mixing between various groundwater bodies, and calculating the groundwater’s residence duration in aquifers^16^. The spatial variances between distinct groundwater end members for the Quaternary aquifer located in Abu Mina basin, which is a part of the Western Nile Delta region, have not been explained by any published studies, despite the significance of water’s isotopic compositions. Therefore, it is essential to comprehend how agricultural activities affect groundwater quality to manage groundwater in desert areas in the interests of sustainability ^18^.

Consequently, combining these approaches will help understand the geochemical processes affecting water chemistry in the investigated region. The overarching goal of this work is to integrate statistical analysis, hydrogeochemical, and stable isotope techniques with conventional tools to outline the geochemical characteristics impacting groundwater quality and to determine the effect of land reclamation on groundwater geochemistry. Irrigation water quality factors that promote sustainable irrigated cultivation in a recently developed land reclamation area were examined.

## Study area characteristics

### Site description

**Fig. 1:**
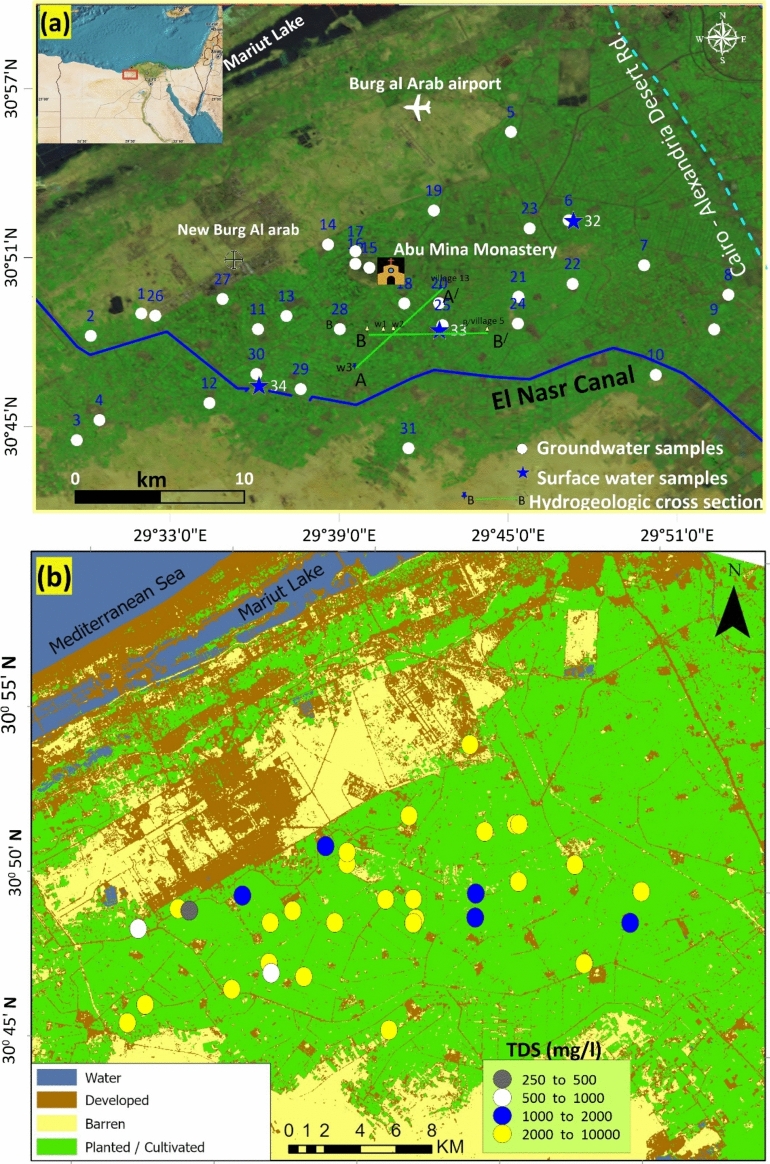
(**a**) A map depicting the location of the investigated region and water sampling locations (initiated using 59); (**b**). Land use and land cover map showing land use class incorporating Total Dissolved Solids (TDS) in the study area (conducted by 60). (Raster data are downloaded from https://earthexplorer.usgs.gov/).

The study area is situated in the northwestern part of the Nile Delta, bordered by the Mariut Tableland to the north and El-Nasr Canal to the south, between longitudes 29° 28′ and 29° 54′ E and latitudes 30° 42′ and 30° 57′ N (Fig. 1). The research covers 1023 km^2^ of the Western Nile Delta’s Abu Mina Basin, a prominent northwest Nile Delta reclamation project.

**Fig. 2:**
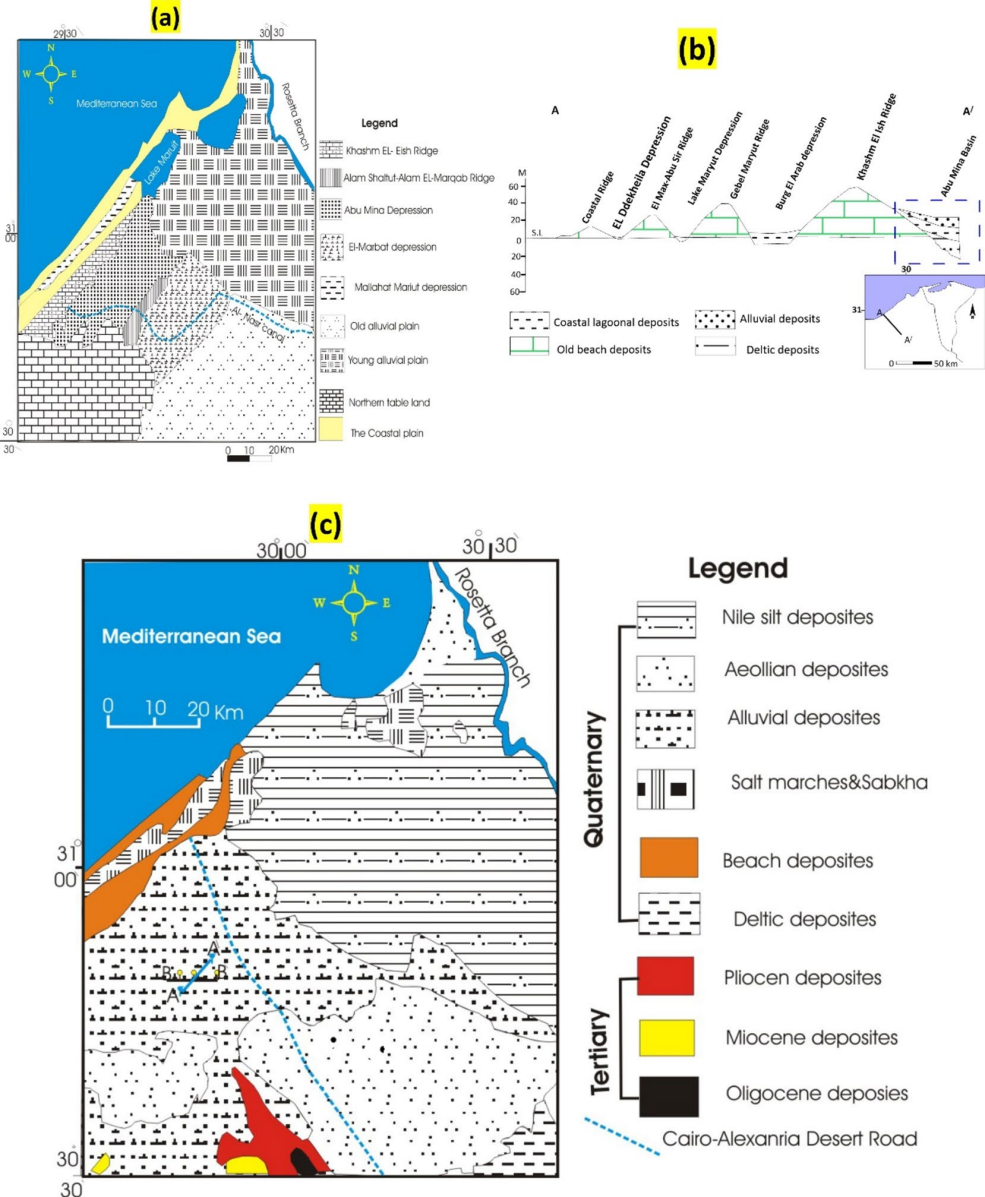
(**a**) Geomorphologic map of the study area and its surroundings (modified after 21); (**b**) Cross-section along Burg El Arab area showing main geomorphic units (after 61); and (**c**) general geologic map of the study area (after 62).

The geomorphology of the studied region is defined by successive ridges running parallel to the current coastline^19^. Four separate ridges have been identified: the coastal ridge (first), El-Max-Abu Sir ridge (second), Gebel Mariut ridge (third), and Khashm El-Ish ridge (fourth) (Figs. 2a,b). These ridges consist of white oolitic and pseudo-oolitic calcareous sand grains and are separated by sabkha-lagoonal depressions^20^. The northern tableland and ridges, composed of Middle Miocene sandy limestone, act as water divides, while Abu Mina and El-Marbat depressions (morphotectonic depressions) serve as water collectors^21^.

### Geological and hydrogeological settings

Geologically, the research region is dominated by the Holocene, Pleistocene, and late Neogene sediments, while the depression areas are primarily composed of alluvial deposits. Tertiary rocks (Pliocene, Miocene, and Oligocene) crop out in the structural plain to the south (Fig. 2c). Pleistocene sediments can be tentatively differentiated into two lateral units. The first unit consists of oolitic limestones, broadly exposed along the Mediterranean Sea coast, comprising detrital limestone associated with calcareous clayey soil^22^. The second unit (40–60 m thick) is mainly exposed in the Abu Mina Basin and consists of fluviomarine facies of sands, clays, and gypsiferous clays.

The surface water system includes the El Nasr Canal, which receives Nile water from the El Nubariya Canal. Over the past ten years, a network of artificially lined canals and open drains has been developed to circulate surface water. El Nasr Canal’s width ranges from 12 to 13 m, with a water depth of about 3.5 m and an estimated seepage rate to groundwater of approximately 0.08 m^3^/km/s^23^.

**Fig. 3:**
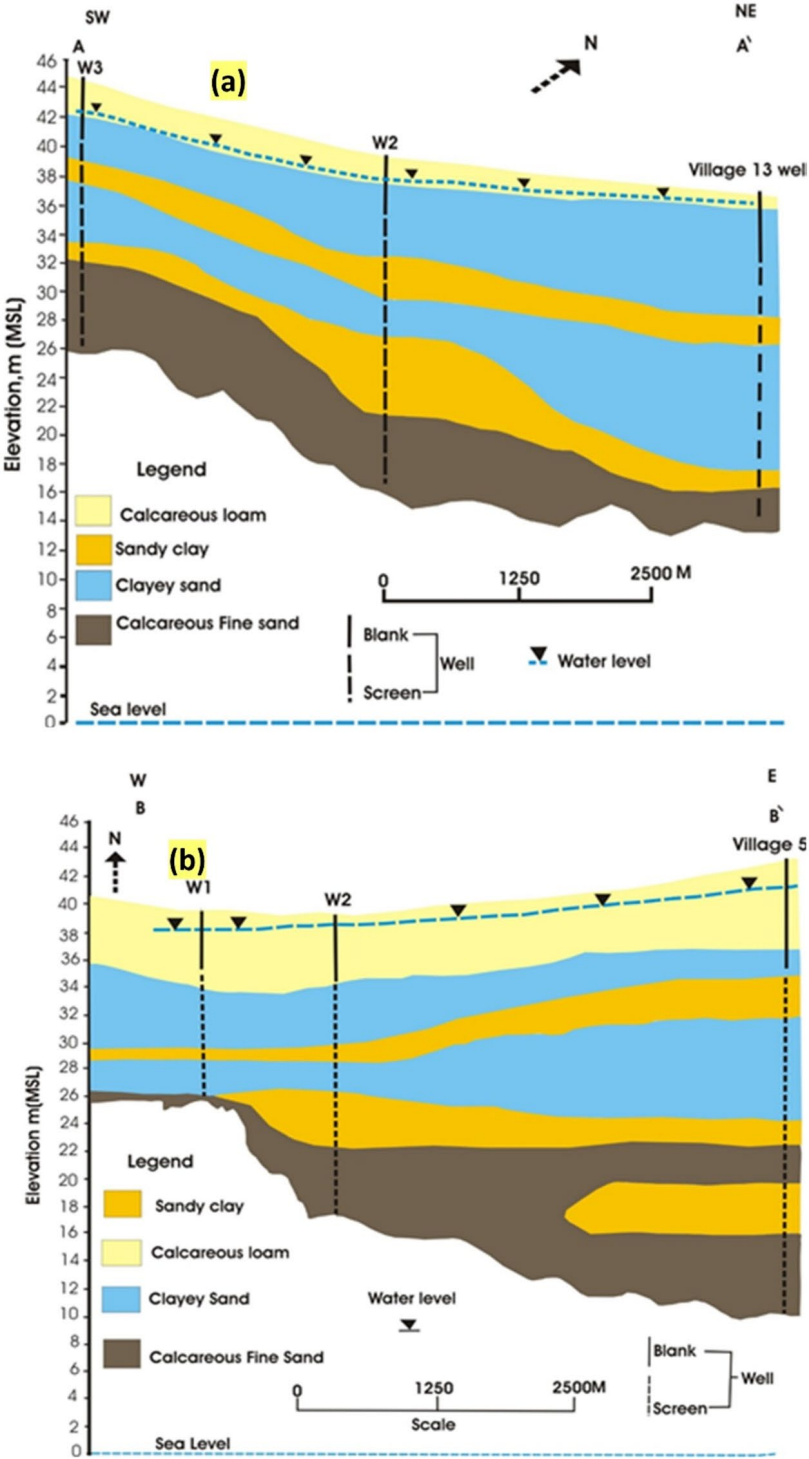
(**a**) Hydrogeologic cross section cuts the study area in a NE-SW direction (**b**) Hydrogeologic cross section cuts the study area in an E-W direction [modified after )25) using (59)].

The research area’s principal aquifers are Quaternary, Pliocene, Miocene, and Oligocene. The current surface water canals, mostly established through Quaternary strata, are hydraulically connected^24^. The predominant water source in the study area is the Pleistocene (Quaternary) aquifer. Water-bearing sediments in the area consist of alternating layers of sand clay and clay sand, determined by drilling (Fig. 3). These layers are capped by a calcareous loamy layer extending across the entire area^25^. Groundwater predominantly exists under semi-confined conditions due to the clastic sediments being covered by Holocene alluvial deposits, with clastic sediment thickness varying from 40 to 60 m.

**Fig. 4:**
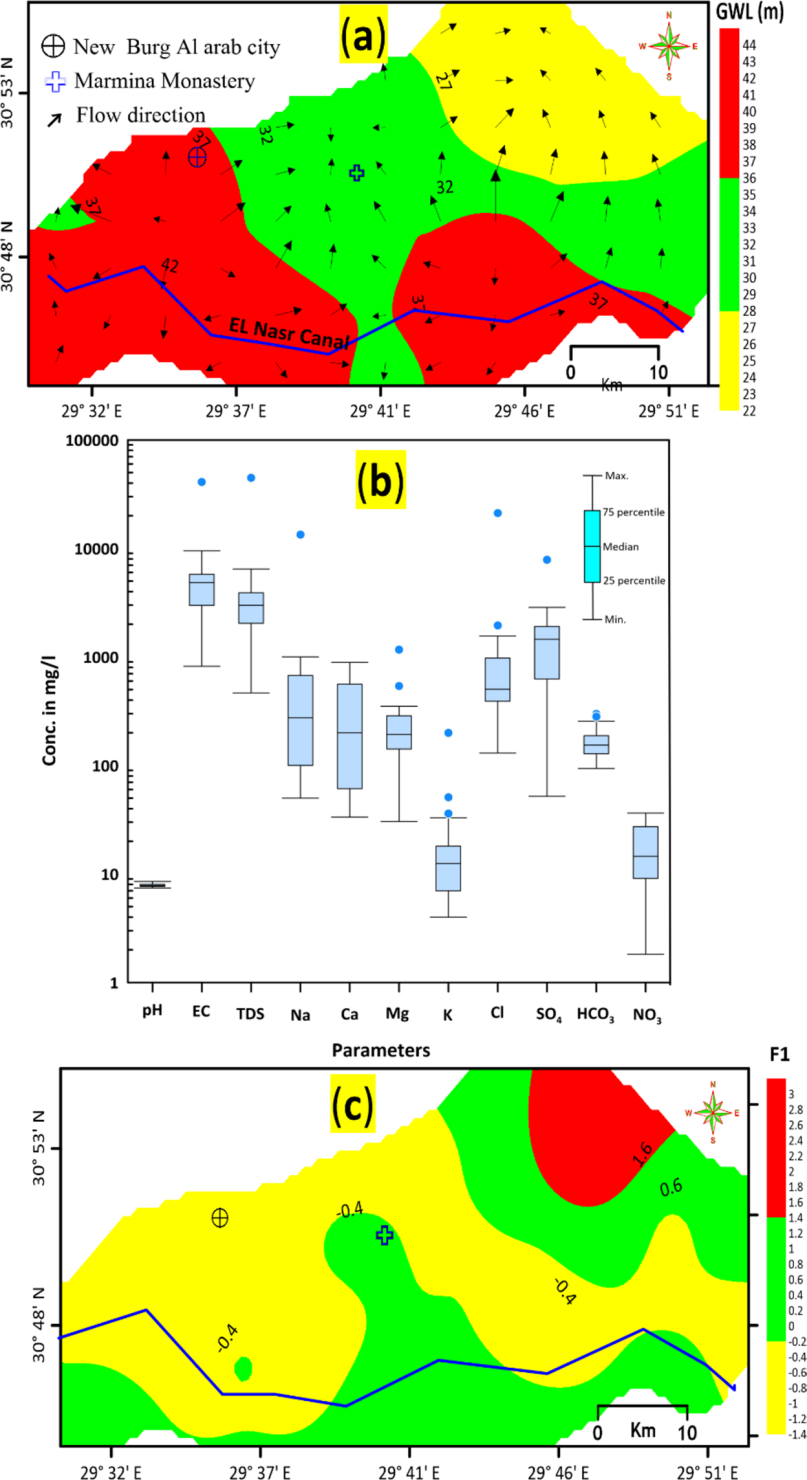
(**a**) Groundwater level map overlayed by vector flow dirction and (**b**) Box and whisker plot of the major ion concentrations in groundwater of Quaternary aquifer (created using 63); (**c**) The first factor score (F1) distribution (a and c has created by (59).

During the 2021 field trip, data were collected from 28 piezometers to study groundwater levels and flow regimes in the Quaternary aquifer. Groundwater primarily flows from the southwest towards the northeast (Fig. 4a). In the study area, the main sources of recharge are infiltration of occasional rainfall, lateral seepage from the El Nasr Canal and its branches, and return flow from excess irrigation. The transmissivity (T) of the Pleistocene aquifer varies from less than 500 m^2^/day to more than 5000 m^2^/day^1^.

## Sampling and analytical techniques

In February 2021, thirty-four water samples were collected from the research area, including one sample from an agricultural drain, two from irrigation canals (El-Nasr canal), and thirty-one groundwater samples representing the Pleistocene aquifer. Fresh aquifer samples were obtained by pumping the boreholes for around 10 min to remove stagnant water. Pre-washed plastic bottles, free of air bubbles, were used to store the collected water samples after filtration with 0.45-micron membrane filters. The physicochemical parameters, including electrical conductivity (EC), temperature (T), and pH, were measured on-site using portable devices.

Total carbonate and bicarbonate concentrations were determined by acid–base titration. The concentrations of Ca^2^⁺, Mg^2^⁺, Na⁺, K⁺, SO₄^2^⁻, and Cl⁻ were determined using an ion chromatography system (Dionex, ICS-1100). Nitrate (NO₃⁻) concentrations were measured using colorimetry with a UV–visible spectrophotometer. Additionally, ionic balance error (IBE) was calculated to check the accuracy of the analysis, with all water samples in the study area showing an IBE within the permissible limit of ± 5%^26^. These chemical analyses were performed in the laboratories of the Desert Research Center (DRC) in Cairo, Egypt. Isotopic analysis (oxygen (δ^1^⁸O) and deuterium (δD)) for eighteen groundwater samples and two surface water samples was conducted at the UC Davis Stable Isotope Facility using headspace equilibration with GasBench-IRMS. The precision of measurement was ± 0.1 ‰ for δ^1^⁸O and ± 1 ‰ for δD. Kyplot software (version 2) was employed to conduct factor analysis and correlation matrix analysis for 11 physiochemical parameters^27^.

## Results and discussion

### Spatial distribution and hydrochemical characteristics

The statistical analysis of the Quaternary aquifer’s results is presented in Table 1 and Fig. 4b. Most parameters show wide ranges and high standard deviations, indicating that processes such as water–rock interaction and anthropogenic effects significantly influence the physicochemical characteristics of the groundwater. The analyzed samples exhibit pH values ranging from neutral to slightly alkaline (7.42–8.53) and EC values from low to high (820–9480 µS/cm). Groundwater salinity varies from fresh to brackish, with a median salinity of 32,988 mg/L. The low salinities near wells 2 and 25 are attributed to natural sporadic replenishment from the El Nasr irrigation canal. The increasing groundwater salinity in the northeastern part of the study area is primarily due to geochemical processes within the aquifer.

**Table 1:** The statistical characteristics of groundwater hydrochemical parameters.

Chemical parameter	Minimum	Maximum	Median	Standard deviation
pH	7.42	8.53	7.88	0.28
EC (µS/cm)	820.00	41,000.00	4850.00	6549.46
TDS (mg/l)	466	44,774	2988	7295.74
Na^+^ (mg/l	50	13,400.00	275.00	2253.04
Ca^2+^ (mg/l)	33.36	889.65	200.17	282.38
Mg^2+^ (mg/l)	30.40	1168.74	192.54	194.39
K^+^ (mg/l)	4.00	200.00	12.50	33.46
Cl^−^ (mg/l)	130.00	21,125.00	503.75	3532.20
SO_4_^2−^ (mg/l)	52.00	7850.00	1456.90	1340.27
HCO_3_^−^ (mg/l	93.90	298.90	153.70	53.16
NO_3_^2−^ (mg/l)	1.82	36.40	14.56	9.95
IBE error (%)	− 2.43	1.79	− 1.30	1.52
δ^2^H (‰)	− 8.39	27.66	22.02	7.44
δ^18^O (‰)	− 1.32	3.52	2.90	1.07

In the study area, geographical distributions of land use/land cover (LULC) for 2022 were created. As depicted in Fig. 1b, the region’s cultivated land was mostly located in the southwest and middle, while its bare land was primarily found in the north and south (the desert region). In the research region, the TDS value varies between 465.60 and 6455.17 mg/l. It is noticeable that all the selected LULC types influenced the spatial depletion in groundwater quality. Nonetheless, there is a correlation between the groundwater salinity distribution and the agricultural distributions in the area. The transition from barren land to development area had the strongest influence overall: it had a significant impact on the change in EC, TDS, and chloride.

**Fig. 5:**
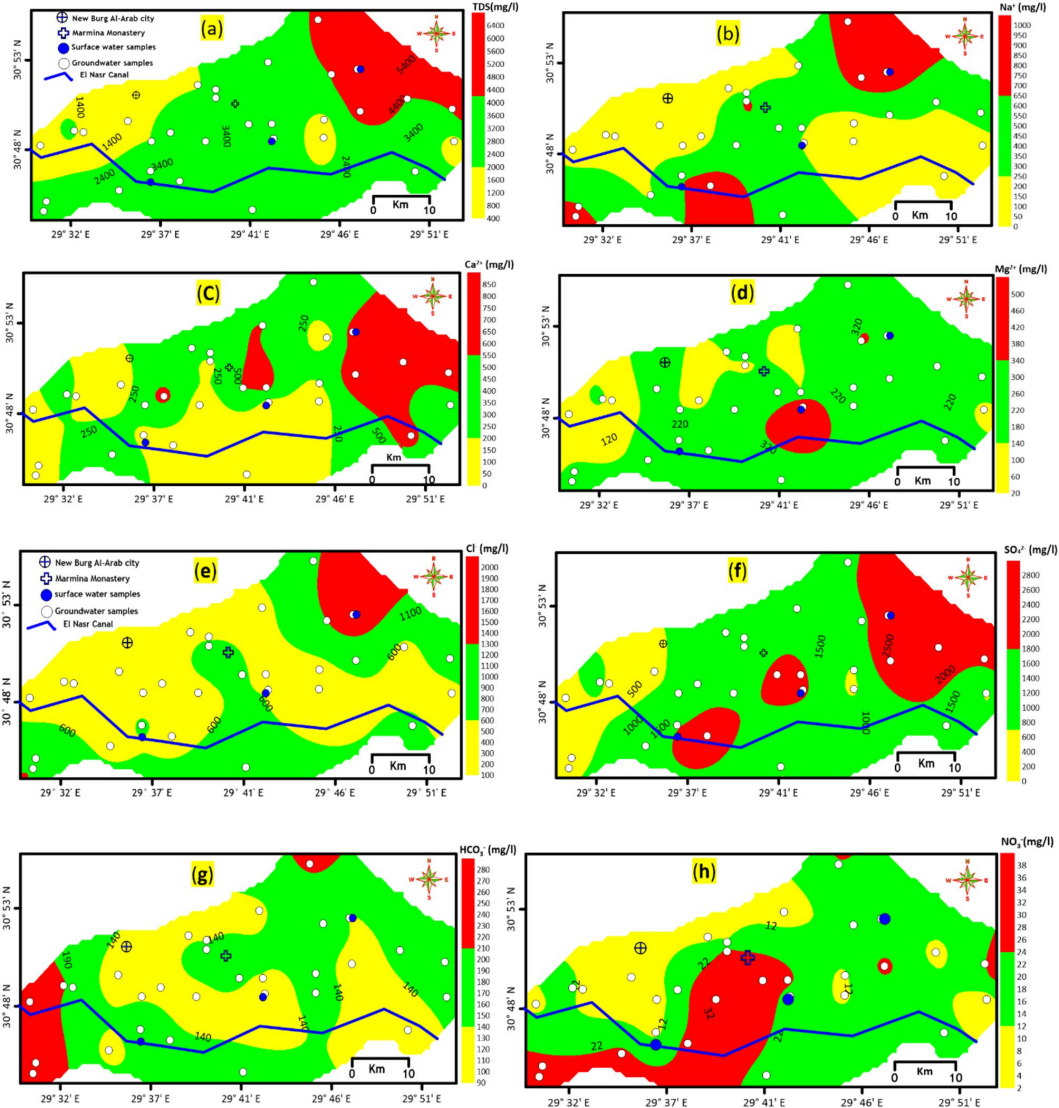
Spatial distribution maps of (**a**) TDS (**b**) Na^+^ (**c**) Ca^2+^ (**d**) Mg^2+^ (**e**) Cl^−^ (**f**) SO_4_^2−^ (**g**) HCO_3_^−^ and (**h**) NO_3_^−^ (data gridding and contouring using 59).

Chemically, the major anions SO₄^2^⁻, Cl⁻, HCO₃⁻, and NO₃⁻ have median concentrations of 1456.90, 503.75, 153.70, and 14.56 mg/L, respectively. The major cations Na⁺, Ca^2^⁺, Mg^2^⁺, and K⁺ have median concentrations of 275, 200.17, 192.54, and 12.50 mg/L, respectively. Generally, the spatial distribution maps of major ions indicate that the concentrations of Na⁺, Ca^2^⁺, Mg^2^⁺, Cl⁻, and SO₄^2^⁻ increase towards the north and northeast of the study area in the direction of increasing total dissolved solids (TDS) and groundwater flow. Conversely, HCO₃⁻ concentrations increase towards the southern part of the study area, indicating the influence of natural recharge from the El-Nasr canal (Figs. 5a–h).

### Constraints from multivariate statistical and geochemical approaches

To comprehend the relationships between the physicochemical components of the groundwater samples, factor analysis (FA) was performed. The analysis included 31 groundwater samples and the variables used were pH, EC, TDS, Ca^2^⁺, Mg^2^⁺, Na⁺, K⁺, HCO₃⁻, SO₄^2^⁻, NO₃⁻, and Cl⁻. The correlation matrix for these 11 variables is shown in Table 2. According to the factor analysis findings, the three most important components explain 71.98% of the total variance (Table 3).

**Table 2:** Pearson correlation matrix of hydrogeochemical parameters determined on groundwater in the study area.

	pH	EC	TDS	Na^+^	Ca^2+^	Mg^2+^	K^+^	Cl^−^	SO_4_^2−^	HCO_3_^−^	NO3^−^
pH	1.00										
EC	− 0.03	1.00									
TDS	− 0.15	**0.96**	1.00								
Na^+^	0.35	**0.78**	**0.65**	1.00							
Ca^2+^	− 0.42	0.38	**0.54**	− 0.18	1.00						
Mg^2+^	− 0.23	**0.61**	**0.64**	0.41	0.00	1.00					
K^+^	0.16	**0.62**	**0.55**	**0.52**	0.31	0.11	1.00				
Cl^−^	0.14	**0.80**	**0.70**	**0.83**	0.09	0.40	**0.61**	1.00			
SO_4_^2−^	− 0.34	**0.73**	**0.86**	0.28	**0.65**	**0.63**	0.28	0.25	1.00		
HCO_3_^−^	**0.68**	− 0.07	− 0.22	0.29	− 0.37	− 0.37	0.32	0.22	**− 0.50**	1.00	
NO_3_^−^	0.11	**0.55**	**0.50**	0.46	0.23	0.19	**0.60**	0.36	0.40	0.06	1.00

Bold text Indicate the correlation coefficients is statistically significant at < 0.05 level.

**Table 3:** Factor loadings of Factor 1, Factor 2 and factor 3.

	Factor1	Factor 2	Factor 3
pH	0.12	− 0.05	− **0.57**
EC	**0.79**	**0.54**	0.20
TDS	**0.69**	**0.63**	0.35
Na^+^	**0.82**	0.20	− 0.12
Ca^2+^	0.07	**0.69**	0.16
Mg^2+^	0.43	0.12	**0.77**
K +	**0.59**	0.39	− 0.30
Cl^−^	**1.00**	0.04	− 0.04
SO_4_^2−^	0.24	**0.79**	**0.57**
HCO_3_^−^	0.20	− 0.14	**− 0.77**
NO_3_^−^	0.34	0.49	− 0.11
Proportion	0.32	0.20	0.19
Cumulative Proportion	0.32	0.53	0.72

Bold text represents significant factor.

Factor 1, revealed that EC, TDS, Na⁺, K⁺, and Cl⁻ accounted for 32.10% of the total variance. The sources of Na⁺ in groundwater in the research region are likely due to silicate weathering, evaporation, and ion exchange processes from the Pliocene clay. Na⁺ and Cl⁻ have strong positive associations (0.83), suggesting a common source. The high TDS content in the groundwater indicated salinity, commonly detected by a high Cl⁻ concentration, which was proportionally associated with cations such as Na⁺. This suggests that Factor 1 depicts contamination by human or natural activity in the newly reclaimed areas of the research region^28,29^. Strong Cl⁻ loading (1.00) suggested the effects of saline water, industrial effluents, and extensive groundwater movement^30,31^. The Factor 1 score distribution pattern in the study area is precisely the same as the TDS distribution map (Fig. 4c), providing additional evidence that the evaporation/rock-water interaction process, which is the prominent factor controlling the overall groundwater chemistry of the study area, has contributed to the high scores of factor 1.

Factor 2, accounted for 20.45% of the total variance and was mainly associated with EC (0.53), TDS (0.63), Ca^2^⁺ (0.68), and SO₄^2^⁻ (0.78). High SO₄^2^⁻ loadings (0.78) were attributed to rock weathering, dissolution, and ion exchange processes affecting groundwater chemistry in the study area. The elevated SO₄^2^⁻ concentrations in groundwater at the newly reclaimed areas may be caused by the use of potassium sulfate fertilizers, the effect of rainfall, and the dissolution of gypsum-anhydrite filling fissures in Pliocene clay^32^.

Factor 3, accounting for about 19.42% of the total variance, includes variables pH, Mg^2^⁺, and HCO₃⁻. The HCO₃⁻ ion is a product of calcite or dolomite dissolution; however, it is not a dominant process in the study area, as indicated by the lowest HCO₃⁻ concentration among the major ions. This suggests the dilution of groundwater and the impact of seepage and infiltration from irrigation water and canals due to new reclamation activities. The higher loading of Mg^2^⁺ and the moderately negative loading of pH suggest that these variables are also influenced by water–rock interactions and ion exchange between Na⁺ and Ca^2^⁺.

### Hydrochemical facies

**Fig. 6:**
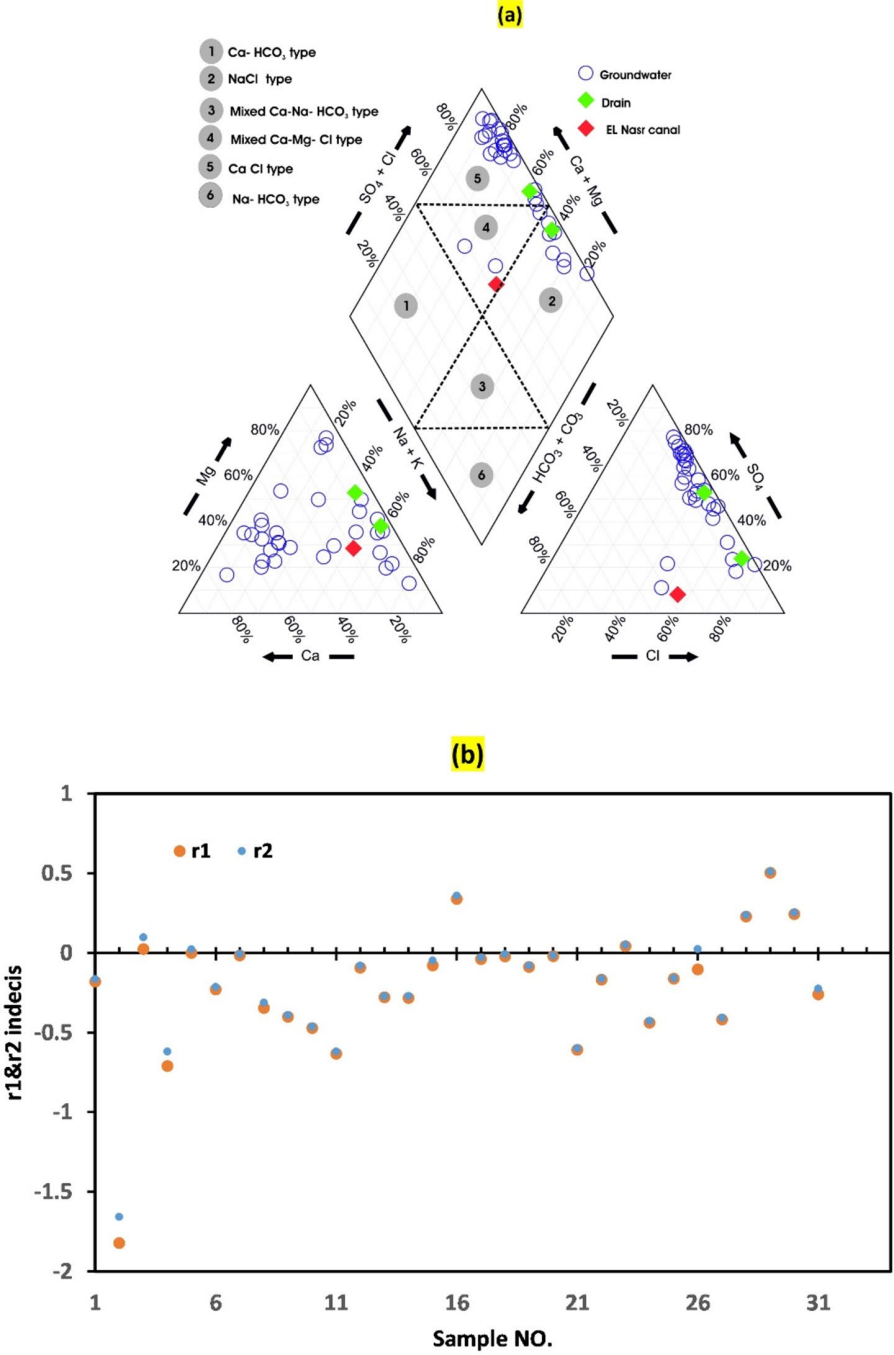
(**a**) Piper diagram showing the various hydrochemical facies in the groundwater (created by 63); (**b**) Base-exchange and meteoric genesis indices (r1&r2) and) showing the various water types and percolation depth.

The results of the chemical analysis of the shallow Quaternary aquifer were plotted on a trilinear Piper diagram^33^. Three hydrochemical facies were identified: the Ca–Cl facies (comprising approximately 74% of the samples), the Na–Cl facies (approximately 19% of the samples), and the mixed Ca–Mg–Cl facies (samples 2, 26, and 34, representing approximately 7% of the samples) (Fig. 6a).Based on the base exchange index (r₁) and the meteoric genesis index (r₂) proposed by^34^ the water type was classified using Eqs. 1 and 2:

1 $$ {\text{Base }} - {\text{ exchange index }}\left( {{\text{r}}1} \right) \, = \, \left( {{\text{Na}}^{ + } - {\text{ Cl}}^{ - } } \right) \, /{\text{ SO}}_{4}^{2 - } $$

2 $$ {\text{Meteoric genesis index }}\left( {{\text{r}}2} \right) \, = \, \left( {{\text{K}}^{ + } + {\text{ Na}}^{ + } } \right) \, - {\text{ Cl}} - \, /{\text{SO}}_{4}^{2 - } $$

The samples were classified as Na⁺–SO₄^2^⁻ water type (r₁ < 1). According to the meteoric genesis index (r₂), the majority of the samples are of deep meteoric water type (r₂ < 1), suggesting that they had longer residence times via deeper percolation (Fig. 6b).

### Hydrogeochemical controls on groundwater chemistry

#### Evaporation process

**Fig. 7:**
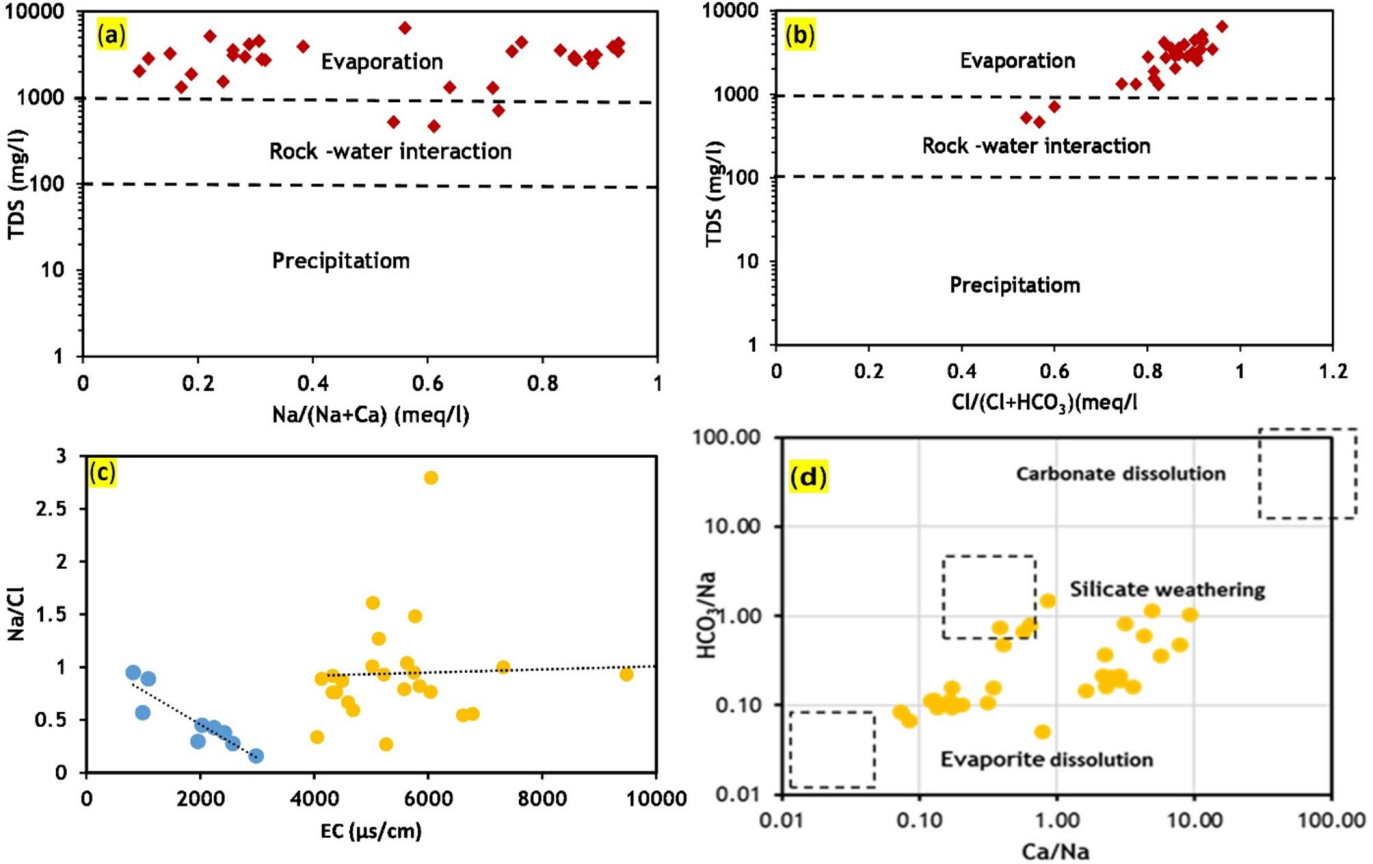
(**a**) and (**b**) Gibbs diagram representing controlling factors of groundwater quality (**c**) Relationship between EC and Na/Cl. (**d**) Na-normalized Ca versus HCO_3_ (carried out with 63).

The Gibbs plot^35^ (Figs. 7a,b) shows that the evaporation process predominantly controls the major ion chemistry of groundwater^36^, with rock-water interaction playing a partial role. The observed increase in total dissolved solids (TDS) against Cl/(Cl + HCO₃) (Fig. 7b) suggests that ion exchange reactions also influence groundwater chemistry. Additionally, heavy fertilizer use, irrigation return flow, and anthropogenic activities contribute to increased salinity through elevated Cl⁻ and Na⁺ levels due to the evaporation process.

To confirm the significance of the evaporation process, a plot of Na/Cl versus electrical conductivity (EC) was prepared (Fig. 7c). The plot reveals that the Na/Cl ratio (in nearly 68% of water samples) remains constant with increasing EC, indicating that evaporation is the dominant process. In contrast, in 32% of water samples, the Na/Cl ratio decreases with increasing EC up to 3000 µS/cm, likely due to Na⁺ depletion via ion exchange processes. In the scatter plot of Ca^2^⁺/Na⁺ versus HCO₃⁻/Na⁺ (Fig. 7d), groundwater samples are primarily dispersed between evaporative dissolution and silicate weathering. This result aligns with the Gibbs plot, suggesting that evaporation is a more significant geochemical factor influencing groundwater chemistry than silicate weathering.

#### Ion exchange process

**Fig. 8:**
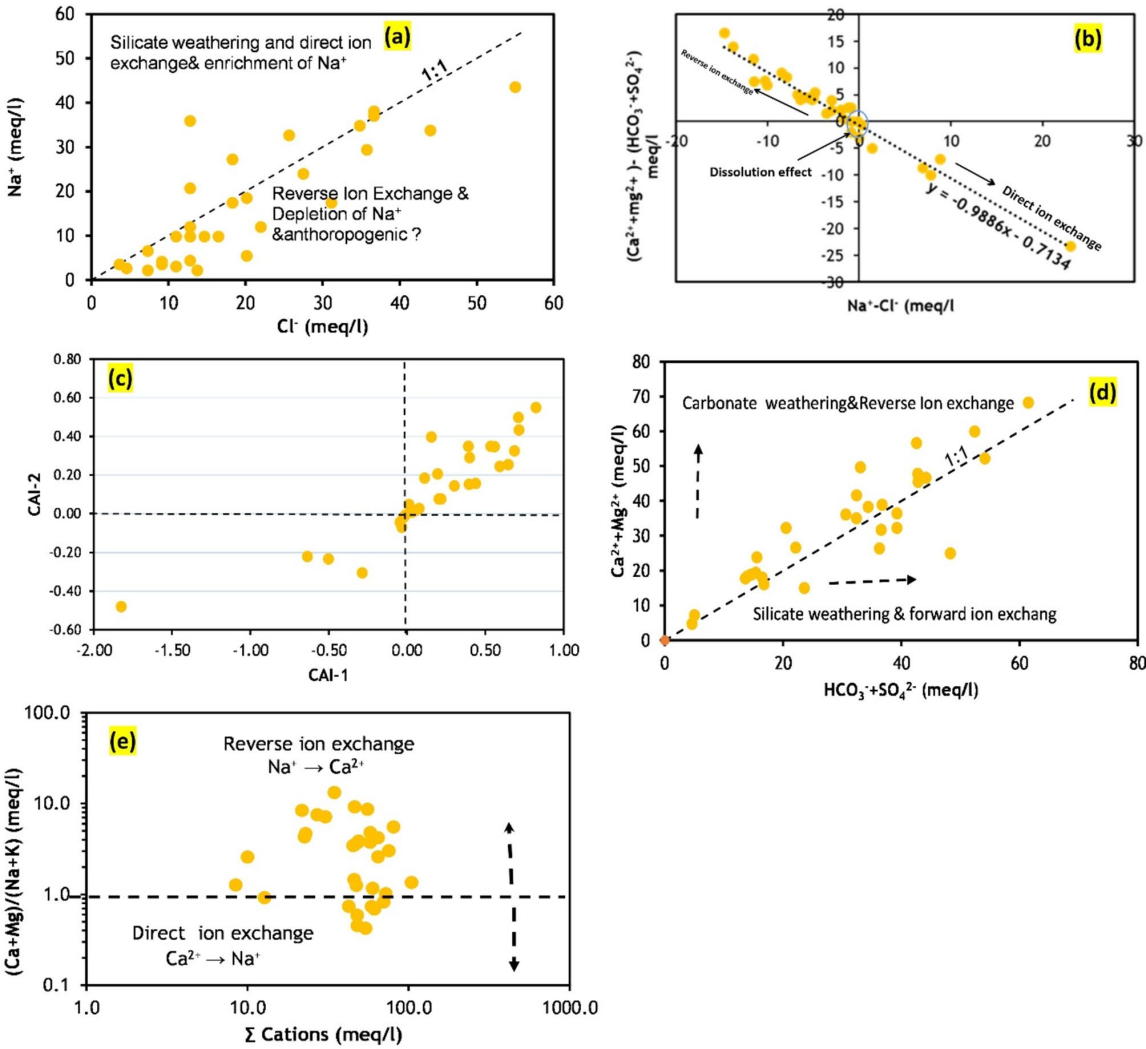
Ions scatter diagram indicating various hydrogeochemical processes of groundwater in the study area (created by 63).

Enrichment or depletion of Na⁺ relative to Cl⁻ indicates ion-exchange processes where Ca^2^⁺ is held in the aquifer matrix, and Na⁺ is released into the groundwater^37^. Figure 8a shows a clear dominance of Cl⁻ over Na⁺ in 76% of samples, indicating Na⁺ depletion due to reverse ion exchange. In 24% of samples, the Na⁺/Cl⁻ ratio is greater than 1, revealing that ion exchange is the main process, replaced by silicate weathering^38^. Human activities and irrigation return flow may augment Cl⁻ in the groundwater, as halite is less prevalent in the investigated region^39^.

A bivariate plot of (Na⁺–Cl⁻) against (Ca^2^⁺ + Mg^2^⁺–HCO₃⁻ + SO₄^2^⁻) (Fig. 8b) shows that most groundwater samples fall on or close to a straight line with a negative slope of − 99.0 and a correlation coefficient of 0.98 (*p* < 0.01), indicating the reverse ion exchange process’s role in Na⁺ and Ca^2^⁺ variations in groundwater^40^.

Chloro-alkaline index calculations (CAI-1 and CAI-2) further interpret the geochemical cation exchange process between groundwater and the aquifer matrix. The indices are computed as follows^41^:

3 $$ {\text{CAI1 }} = {\text{ C l}}^{ - } \left( {{\text{Na}}^{ + } + {\text{K}} + } \right)/{\text{Cl}}^{ - } $$

4 $$ {\text{CAI }}2 \, = {\text{ Cl}}^{ - } \left( {{\text{Na}}^{ + } + {\text{K}}^{ + } } \right)/{\text{SO}}_{4}^{2 - } + {\text{HCO}}_{3}^{ - } + {\text{CO}}_{3}^{2 - } + {\text{NO}}_{3}^{ - } $$

where the units of ions are in meq/L.

A negative CAI value implies that ion exchange is the dominant process, whereas a positive value indicates reverse ion exchange predominates. As shown in Fig. 8c, 77% of samples have positive CAI values, suggesting a reverse ion exchange reaction where Ca^2^⁺ and Mg^2^⁺ in the aquifer matrix replace Na⁺ and K⁺ in groundwater. In contrast, 23% of samples have negative CAI values, indicating forward ion exchange where Na⁺ is emitted from the aquifer matrix, and Ca^2^⁺ is absorbed:

5 $$ {\text{2NaX }} + {\text{ Ca}}^{{{2} + }} \to {\text{2Na}}^{ + } + {\text{ CaX2}}\quad{\text{forward ion exchange}} $$

6 $$ {\text{CaX}}_{{2}} + {\text{ 2Na}}^{ + } \to {\text{Ca}}^{{{2} + }} + {\text{ 2NaX}}\quad{\text{reverse ion exchange}} $$

where X = aquifer solid, Ca^2+^, Na^+^, and Mg^2+^ are Calcium, sodium, and magnesium ions, respectively.

Moreover, a plot of total Ca^2^⁺ + Mg^2^⁺ ions versus HCO₃⁻ + SO₄^2^⁻ ions indicates that the reverse ion exchange process predominates in the aquifer, with 76% of samples showing a notable increase in Ca^2^⁺ + Mg^2^⁺ ions compared to HCO₃⁻ + SO₄^2^⁻ ions ^42^ (Fig. 8d). A bivariate plot of the (Ca^2^⁺ + Mg^2^⁺)/(Na⁺ + K⁺) ratio against total cations (Fig. 8e) shows that 73% of samples fall within the reverse ion exchange region, although a few points (27% of samples) suggest direct ion exchange predominates ^43,44^. Consequently, both forward and reverse ion exchange reactions govern the hydrochemistry of the aquifer in the studied region.

#### Silicate and carbonate weathering implications

**Fig. 9:**
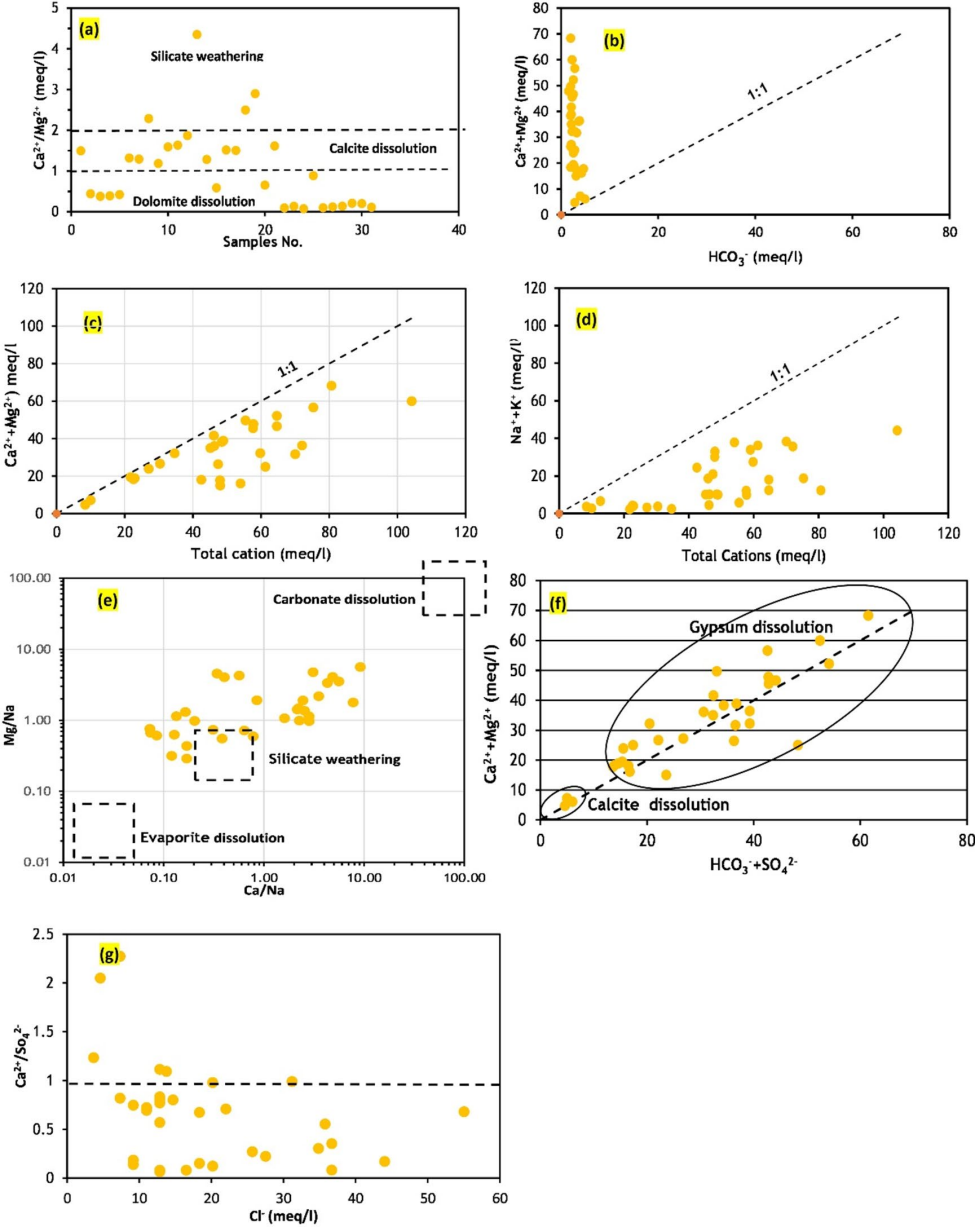
Equiline diagrams for groundwater showing the correlation of major ions to discriminate the entire processes act in the aquifer (created by 63).

Table 4 demonstrates that the values of Cl/Σ anions, Na⁺/Cl⁻, Na⁺/(Na⁺ + Ca^2^⁺), and Mg^2^⁺/(Ca^2^⁺ + Mg^2^⁺) are less than 1, highlighting the impact of silicate weathering on groundwater chemistry in the area^45^. Silicate weathering significantly increases SO₄^2^⁻ and HCO₃⁻ ions (24% of samples) compared to Ca^2^⁺ and Mg^2^⁺ ions (Fig. 8d). The Ca^2^⁺/Mg^2^⁺ ratio was less than 1 in most samples (52%), and between 1 and 2 in 33% of samples, indicating that dolomite and calcite dissolution contribute to high Ca^2^⁺ and Mg^2^⁺ levels, followed by silicate mineral dissolution^46^ (Fig. 9a). All water samples showed Ca^2^⁺ and Mg^2^⁺ concentrations above the 1:1 aquiline in the scatter plot of (Ca^2^⁺ + Mg^2^⁺) versus (HCO₃⁻), indicating that silicate weathering is more prevalent than carbonate weathering^47^ (Fig. 9b). The high concentrations of Ca^2^⁺ and Mg^2^⁺ in the groundwater are likely due to silicate mineral dissolution, as 52% of samples have an Mg^2^⁺/(Mg^2^⁺ + Ca^2^⁺) ratio greater than 0.5^48,49^.

**Table 4:** Ratio of ion exchange reaction and silicate weathering in Quaternary aquifer.

Parameter	Minimum	Maximum	average
Na^+^/(Na^+^ + Ca^2+^)	0.10	0.93	0.54
CAI1	− 1.82	0.82	0.16
CAI2	− 0.48	0.57	0.13
Cl^−^/∑Anions	0.19	0.72	0.39
Mg^2+^/(Ca^2+^ + Mg^2+^)	0.19	0.93	0.59
r1	− 1.82	0.50	− 0.24
r2	− 1.66	0.51	− 0.21
Na^+^/Cl^−^	0.16	2.80	0.82

Scatter plots of Ca^2^⁺ + Mg^2^⁺ versus total cations and Na⁺ + K⁺ versus total cations show that all water samples are plotted below the 1:1 equiline (Fig. 9c,d), indicating that silicate weathering mainly contributes Na⁺ and K⁺ ions to the water system ^50^. In bivariate plots (Fig. 7c, 9e), water samples predominantly scatter between the weathering of silicate and carbonate minerals’ dissolution, suggesting significant contributions from both processes to groundwater chemistry.

Approximately 10% of sampling points fall on the 1:1 line of Ca^2^⁺ + Mg^2^⁺ versus HCO₃⁻ + SO₄^2^⁻ scatter plot with HCO₃⁻ + SO₄^2^⁻ less than 10 meq/L, suggesting calcite dissolution (Fig. 9f). Around 90% of samples with HCO₃⁻ + SO₄^2^⁻ greater than 10 meq/L in the scatter plot of Ca^2^⁺ + Mg^2^⁺ versus HCO₃⁻ + SO₄^2^⁻ are affected by gypsum dissolution. Samples falling above the unity line in the Ca^2^⁺/SO₄^2^⁻ molar ratio versus Cl⁻ (meq/L) plot had minimal influence from gypsum dissolution^5^ (Fig. 9g).

## Indicators from stable isotopes for recharge sources and salinization

**Fig. 10:**
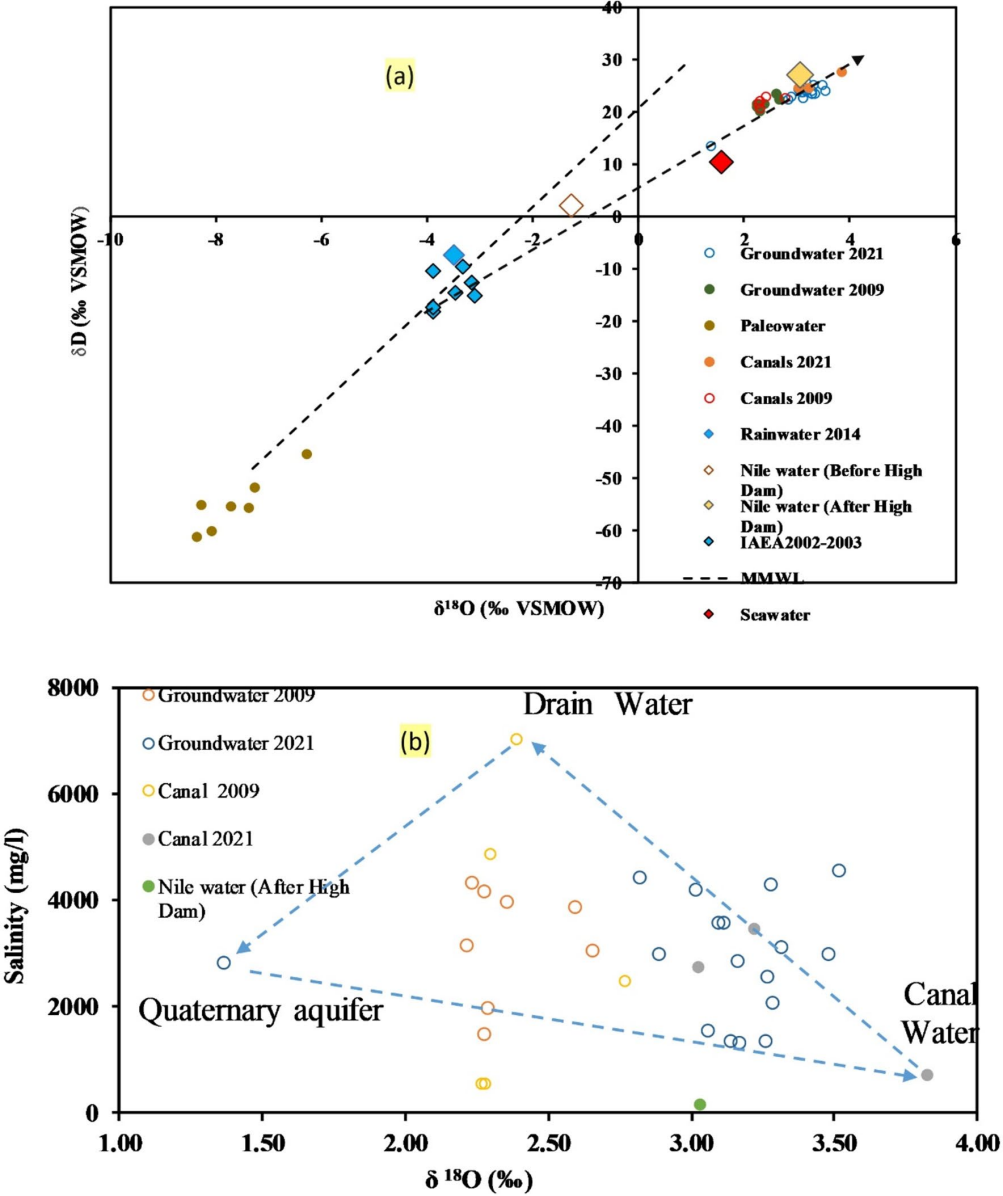
(**a**) ^δ2^H‰ vs ^δ18^O‰ values for water resources; (**b**) Change of δ^18^O‰ vs Cl (mg/l) values for water resources associated with different salinization processes (created by 63).

The stable isotopic content of groundwater provides insights into recharge and mixing sources^51^. Isotopes (δ^1^⁸O, δ^2^H) are part of water molecules unaffected by water–rock interaction^52^. Isotopic signatures change due to evaporation, Rayleigh distillation, and mixing with water of different signatures^53^. The δ^1^⁸O concentrations in groundwater range from − 1.31‰ (well 15) to + 3.53‰ (well 8), while δ^2^H values range from − 8.38‰ (well 8) to 25.48‰ (well 13). In Fig. 10a, δ^1^⁸O is plotted against δ^2^H, aligned with the Global Meteoric Water Line (GMWL, Craig 1961) and the Mediterranean Meteoric Water Line (MMWL), using rainwater, seawater, canal water, and paleowater samples as end members to evaluate the influence of various processes on groundwater quality. The rainwater samples are plotted close to the GMWL and MMWL^54^, while groundwater samples (except well 15) collected in 2009 and 2020 are situated near the canal water on the evaporation trend line extending from rainwater. In Fig. 10b, the groundwater samples have been plotted between three end members: the rainwater, the canal water, and the Quaternary groundwater, represented by sample No.1, indicating the prime source of recharge is return irrigations flux and seepage from the canals. Sample No. 15 is plotted close to the seawater indicating possible mixing with the seawater.

## Irrigation water quality

**Fig. 11:**
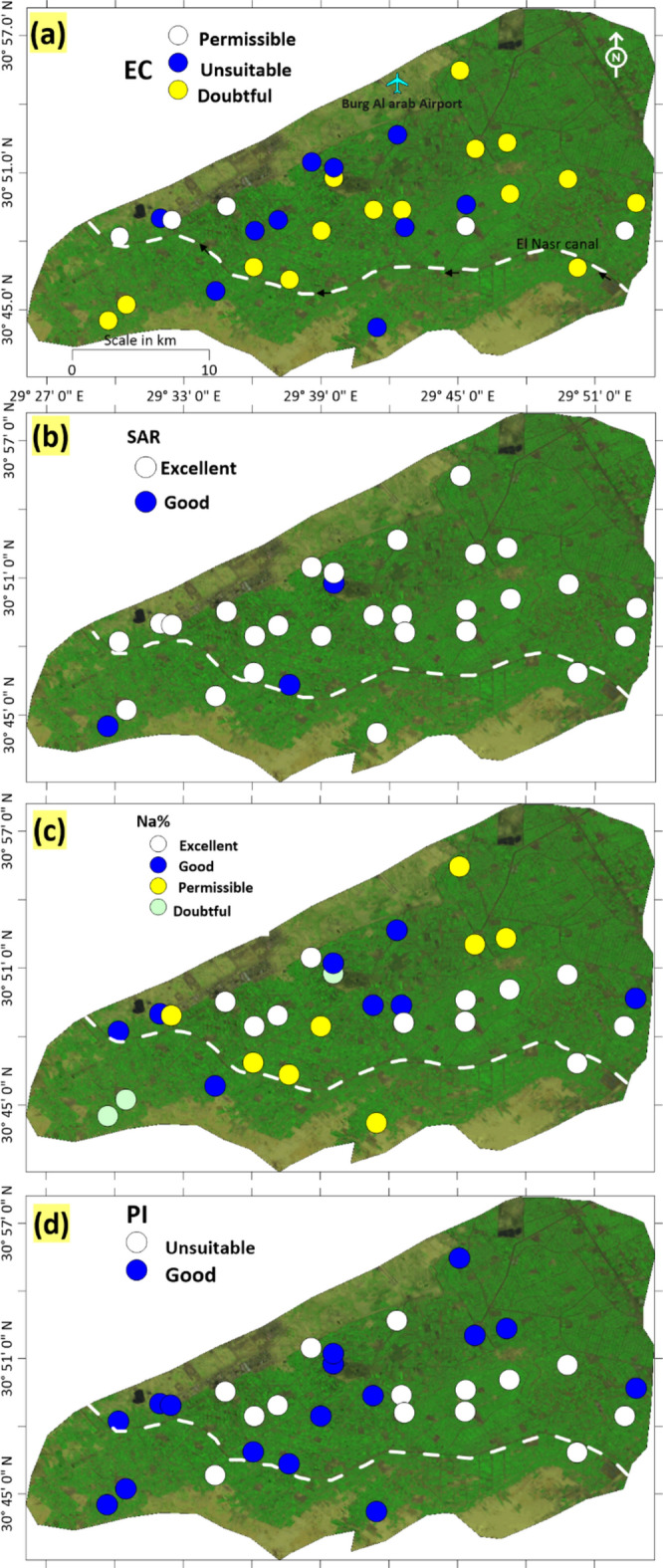
Spatial distribution maps of irrigation water quality in the study area. (**a**) EC (**b**) SAR (**c**) Na% and (**d**) PI (created by 59).

The increased salinity of irrigation water has a negative effect on the soil and plants. The mineral salts that exist in the irrigation water can create changes in the structure of the soil, affecting its permeability and aeration, which leads to a disruption in the growth of plants^55^. The rise in the salinity of irrigation water has an adverse impact on the soil and plants. We assessed the proper suitability of groundwater for irrigation in the research region using qualitative indicators such as sodium adsorption ratio (SAR), electrical conductivity (EC), permeability index (PI), sodium percentage Na (%), residual sodium carbonate (RSC), and spatial representations. All the concentrations for these criteria are measured in meq/l (Table 5). In general, the spatial distribution map of groundwater acceptability for irrigation in the area under consideration shows that groundwater from the majority of wells is suitable for irrigation purposes (Table 5 & Figs.11a–d). Nevertheless, it is imperative to consider the salinization processes of groundwater. Suitable irrigation methods should be used in this area and similar areas with limited recharge to prevent the depletion of the aquifer, which results from over pumping.

**Table 5:** Irrigation water quality parameters from Quaternary aquifer based on EC, SAR, Na%, RSC, and PI.

Parameter	References	Formula Adopted	Categories	Ranges	% samples
EC	^56^		Excellent	< 250	–
			Good	250–750	–
			Permissible	750–2250	16.7
			Doubtful	2250–5000	13.3
			Unsuitable	> 5000	50.0
SAR	^57^	$\frac{{Na^{ + } }}{{\frac{{\sqrt {Ca^{2 + } + Mg^{2 + } } }}{2}}}$	Excellent	0–10	90
			Good	10–18	10
			Doubtful	18–26	–
			Unsuitable	> 26	–
Na %	^56^	$\frac{{{\text{Na}}^{ + } + {\text{K}}^{ + } }}{{{\text{Ca}}^{2 + } + {\text{Mg}}^{2 + } + {\text{Na}}^{ + } + {\text{K}}^{ + } }} \times 100$	Excellent	0–20	36.6
			Good	20–40	26.7
			Permissible	40–60	26.7
			Doubtful	60–80	10.0
			Unsuitable	80–100	–
RSC	^57^	$\left( {{\text{CO}}_{{3}}^{{{2} - }} + {\text{HCO}}_{{3}}^{ - } } \right) - \left( {{\text{Ca}}^{{{2} + }} + {\text{Mg}}^{{{2} + }} } \right)$	Good	< 1.25	100
			Medium	1.25–2.5	–
			bad	> 2.5	–
PI	^58^	$\frac{{{\text{Na}}^{ + } + \sqrt {{\text{HCO}}_{3}^{ - } } }}{{({\text{Na}}^{ + } + {\text{Ca}}^{2 + } + {\text{Mg}}^{2 + } )}} \times 100$	Unsuitable	0–25	46.7
			Good	25–75	53.3
			Excellent	≥ 75	–

## Conclusion and recommendations

Based on a comprehensive analysis of various water quality parameters and inorganic elements in the Abu mina basin, NW coast of Egypt, several key conclusions can be drawn, as follows:

The Pleistocene alluvium deposits host the main aquifer in the study area, namely the Quaternary aquifer. The results of a hydrogeochemical and isotopic investigation of Quaternary aquifer in the new land reclamation area of the northwestern Nile Delta, Egypt, are utilized to highlight the geochemical properties, recharge sources, and potential key processes controlling groundwater chemistry. The groundwater of the Quaternary aquifer is meteoric in origin. Groundwater salinity ranged from 465.60 to 6455.18 mg/l (fresh to brackish water), with slightly alkaline (7.42 < pH < 8.15). The greater range of total dissolved solids (TDS) in shallow groundwater suggests that the quality of shallow groundwater is affected by evaporation, infiltration of irrigated water, and convection of saline water from the deeper aquifer. The calculated meteoric genesis index (r^2^) indicates the dominance of deep meteoric water percolation effects on the groundwater chemistry of the Pleistocene aquifer. The concentration patterns of cations and anions demonstrate a consistent trend, with concentrations increasing towards the northeast.

In this research, the main anion sequence of groundwater is SO₄^2^⁻ > Cl⁻ > HCO₃⁻ > NO₃⁻, respectively. The main cation sequence is Na⁺ > Ca^2^⁺ > Mg^2^⁺ > K⁺, respectively. Most water samples belong to the Ca–Cl facies, Na-Cl facies, and mixed Ca–Mg–Cl facies, in decreasing order of abundance. Multivariate statistical analysis (FA) is a well-established methodology that was implemented for classifying waters and identifying critical components influencing water quality. The groundwater hydrochemistry can be explained by three factors, which together account for 71.98% of the total variance. According to the rotating components matrix (F1–F3), the chemistry of groundwater is principally affected by evaporation, ion exchange reactions, and human activities. The Quaternary aquifer is salinized by natural and agricultural processes, including evaporation, ion exchange, and carbonate–silicate dissolution along groundwater flow and irrigation return flow. In general, surface water irrigation practices have affected the hydrogeochemical properties of groundwater. The study of environmental isotopes confirms this.

The isotopic data demonstrated that the surface water samples (δ ^18^O = 3.82‰ and δ ^2^ H = 27.65‰)) have significant isotopic content relative to the recent Nile River water (δ ^18^O = 3.03‰ and δ ^2^ H = 27‰). This indicates that there is an evaporation process occurring in surface water bodies throughout its flow, resulting in an increase in isotopic content. The groundwater isotopic content generally increased to its maximum values in response to the recent Nile water, canals, and drain water, which reflects the mixed condition of groundwater that was recharged post the construction of the High Dam.

The depletion of δ^1^⁸O and δ^2^H values in groundwater samples indicates that the main sources of recharge are seepage from irrigation canals and possible mixing with seawater. Our research may prove helpful for the sustainable management of in desert reclamation projects in arid regions and its impact on groundwater quality. Understanding the most important hydrogeochemical processes is essential for future groundwater management and environmental protection.

All groundwater samples are appropriate for agricultural irrigation based on the Sodium Adsorption Ratio (SAR), Permeability Index (PI), Percent Sodium (%Na), and Residual Sodium Carbonate (RSC). While certain elements fall within safe limits for irrigation, others exceed permissible concentrations (as EC), posing risks to soil and plant health. This investigation will offer policymakers the necessary information to ensure the sustainable administration of groundwater resources in the reclamation area.

The current work could be enhanced by.

- Employing a groundwater flow model to provide a complete picture of water flow and groundwater quality via an aquifer.
- More detailed land use maps are created by using high-definition satellite data.
- Furthermore, the construction of an accurate water quality index based on numerous variables and water parameters could be important for improved understanding of water sources and their mixing patterns, particularly in shallow alluvial aquifers under arid conditions.
- Sustaining groundwater quality management and protection requires ongoing monitoring of groundwater quality.

## Data Availability

The datasets used and/or analyzed during the current study are available from the corresponding author on request.

## References

[1] Salem, Z. E. *et al.* Hydrogeochemical analysis and evaluation of groundwater in the reclaimed small basin of Abu Mina, Egypt. *Hydrogeol. J.* **23**(8), 1781 (2015).

[2] Ahmed, M. *et al.* Isotopic composition of groundwater resources in arid environments. *J. Hydrol.* **609**, 127773 (2022).

[3] Omar, M. E. D. *et al.* Impacts of climate change on water quantity, water salinity, food security, and socioeconomy in Egypt. *Water Sci. Eng.* **14**, 17–27 (2021).

[4] Ammar, S. B. *et al.* Using geochemical and isotopic tracers to characterize groundwater dynamics and salinity sources in the Wadi Guenniche coastal plain in northern Tunisia. *J. Arid Environ.* **178**, 104150 (2020).

[5] Karunanidhi, D. *et al.* Appraisal of subsurface hydrogeochemical processes in a geologically heterogeneous semi-arid region of south India based on mass transfer and fuzzy comprehensive modeling. *Environ. Geochem. Health* **43**(2), 1–20 (2020). 32719980 10.1007/s10653-020-00676-2

[6] Keesari, T. & Dauji, S. Groundwater salinization processes: Pitfalls of inferences from Na+/Cl− versus Cl− correlation plots. *Environ. Geochem. Health* **43**(2), 949–969 (2020). 32588160 10.1007/s10653-020-00622-2

[7] Sunkari, E. D. *et al.* Geochemical evolution and tracing of groundwater salinization using different ionic ratios, multivariate statistical and geochemical modeling approaches in a typical semi-arid basin. *J. Contam. Hydrol.* **236**, 103742 (2021). 33246311 10.1016/j.jconhyd.2020.103742

[8] Wang, W. *et al.* Water quality and interaction between groundwater and surface water impacted by agricultural activities in an oasis-desert region. *J. Hydrol.* **617**, 128937 (2023).

[9] Alshrabsy, A. N. *et al.* Assessment of irrigation water quality in the reclaimed lands in the north of Dakahlia Governorate. *J. Soil Sci. Agric. Eng.* **15**(3), 51–65 (2024).

[10] Hussain, G. *et al.* Guidelines for irrigation water quality and water management in the Kingdom of Saudi Arabia: An overview. *J. Appl. Sci.*10.3923/jas.2010.79.96 (2010). 10.3923/jas.2010.79.96

[11] Salem, Z. E. S. & El-horiny, M. M. Hydrogeochemical evaluation of calcareous eolianite aquifer with saline soil in a semiarid area. *Environ. Sci. Pollut. Res.* **21**, 8294–8314 (2014). 10.1007/s11356-014-2735-9 24691931

[12] Yang, N. *et al.* Application of multiple approaches to investigate the hydrochemistry evolution of groundwater in an arid region: Nomhon. *Northwestern China. Water* **10**(11), 1667 (2018).

[13] Jampani, M. *et al.* (Hydrogeochemical and mixing processes controlling groundwater chemistry in a wastewater irrigated agricultural system of India. *Chemosphere* **239**, 124741 (2020). 31518921 10.1016/j.chemosphere.2019.124741

[14] Sarker, M. M. *et al.* Identifying the major hydrogeochemical factors governing groundwater chemistry in the coastal aquifers of Southwest Bangladesh using statistical analysis. *Hydrology* **9**(2), 20 (2022).

[15] Salem, Z. E. S. *et al.* Oxygen and hydrogen stable isotopes as recharge indicators, Central Nile Delta Quaternary aquifer, Egypt. *J. King Saud Univ. Sci.* **34**(3), 101834 (2022).

[16] Trabelsi, R. *et al.* Assessment of geochemical processes in the shared groundwater resources of the Taoudeni aquifer system (Sahel region, Africa). *Hydrogeol. J.* **32**(1), 167–188 (2024).

[17] Jeelani, G. *et al.* Use of stable water isotopes to identify and estimate the sources of groundwater recharge in an alluvial aquifer of Upper Jhelum Basin (UJB), western Himalayas. *Hydrol. Sci. J.* **66**, 2330–2339 (2021).

[18] Hamidi, M. D. *et al.* Investigating groundwater recharge using hydrogen and oxygen stable isotopes in Kabul city, a semi-arid region. *J. Hydrol* **626**, 130187 (2023).

[19] El-Bayomi, G. M. Coastal environmental changes along the north-western coast of Egypt. A case study from Alexandria to El-Alamein coast. Forum Geographic. *Stud. Cercet. Geogr. Med.* **8**, 14–22 (2009).

[20] Embabi, N.S. The Geomorphology of Egypt: Landforms and Evaluation. The Egyptian Geographical Society, The Nile Valley and Western Desert, p. 447. (2004).

[21] Abdel-Baki, A. M. A. Hydrogeological and Hydrochemical Studies on the Area West of Rosetta Branch and South of El-Nasr Canal. Ph. D. Thesis, Ain Shams University. (1983).

[22] Abdel Mogheeth, SM. Hydrogeochemical studies of Bur El-Arab and vicinities. MSc Thesis, Ain Shams University, Egypt (1968).

[23] Salem, Z. E. S. & Osman, O. M. Use of major ions to evaluate the hydrogeochemistry of groundwater influenced by reclamation and seawater intrusion, West Nile Delta, Egypt. *Environ. Sci. Pollut. Res.* **24**, 3675–3704 (2017). 10.1007/s11356-016-8056-4 27888479

[24] Sharaky, A. M.*et al*. Hydrogeochemistry of groundwater in the Western Nile Delta aquifers, Egypt. In 2nd International Conference on the Geology of Tethys. Cairo University. (2007), Vol. 19, No. 21, pp. 1–23.

[25] El-Sayed, S. A. *et al.* Groundwater table rise in northwest Nile Delta: Problems and recommendations. *J. Radiat. Res. Appl. Sci.* **5**(1), 141–171 (2012).

[26] Appelo, C. A. J. & Postma, D. *Geochemistry, groundwater and pollution* 2nd edn. (CRC Press, 2005).

[27] Yoshioka, K., KyPlot Program Version 2.0. www.phy.gonzaga.edu, Accessed date:3 April 2007. (2001).

[28] Ahmed, A. A. & Ali, M. H. Hydrochemical evolution and variation of groundwater and its environmental impact at Sohag, Egypt. *Arab. J. Geosci.* **4**(3), 339–352 (2011).

[29] Subba Rao, N. *et al.* Chemical characteristics of groundwater and assessment of groundwater quality in Varaha River Basin, Visakhapatnam District, Andhra Pradesh, India. *Environ. Monit. Assess.* **184**, 5189–5214 (2012). 21931947 10.1007/s10661-011-2333-y

[30] Kumar, D. & Alappat, B. J. Analysis of leachate pollution index and formulation of sub-leachate pollution indices. *Waste Manag. Res.* **23**(3), 230–239 (2005). 15988942 10.1177/0734242X05054875

[31] Venkatramanan, S. *et al.* Factors controlling groundwater quality in the Yeonjegu District of Busan City, Korea, using the hydrogeochemical processes and fuzzy GIS. *Environ. Sci. Pollut. Res.* **24**, 23679–23693 (2017). 10.1007/s11356-017-9990-5 28861839

[32] Redwan, M. & Moneim, A. Factors controlling groundwater hydrogeochemistry in the area west of Tahta, Sohag. *Upper Egypt. J. Afr. Earth Sci.* **118**, 328–338 (2015).

[33] Piper, A. M. A graphic procedure in the geochemical interpretation of water-analyses. *EOS Trans. Am. Geophys. Union* **25**(6), 914–928 (1944).

[34] Soltan, M. E. Evaluation of groundwater quality in Dakhla oasis (Egyptian western desert). *Environ. Monit. Assess.* **57**, 157–168 (1999).

[35] Gibbs, R. J. Mechanisms controlling world water chemistry. *Sci. J.* **170**, 1088–1090 (1970). 10.1126/science.170.3962.1088 17777828

[36] Li, X. *et al.* Hydrochemical characteristic and interaction process of surface and groundwater in mid-lower reach of Hanjiang River, China. *Environ. Earth Sci.* **75**, 418 (2016).

[37] Rajimohan, N. & Elango, L. Identification and evaluation of hydrogeochemical processes in the groundwater environment in an area of the Palar and Cheyyar river basins, southern India. *Environ. Geol.* **46**, 47–61 (2004).

[38] Embaby, A. & Ali, M. Hydrogeochemical processes controlling groundwater in Western Sohag Governorate, Upper Egypt. *Arab. J. Geosci.* **14**(9), 789 (2021).

[39] Mohallel, S. A. Hydrogeochemical Study for sustainable development in Wadi El-Mathula, East Qift city, Eastern Desert. *Egypt. Int. J. Environ.* **9**(2), 60–82 (2020).

[40] Jia, H. *et al.* Alterations to groundwater chemistry due to modern water transfer for irrigation over decades. *Sci. Total Environ.* **717**, 137170 (2020). 32062271 10.1016/j.scitotenv.2020.137170

[41] Schoeller, H. Qualitative evaluation of groundwater resources. Methods and techniques of groundwater investigations and development. UNESCO, 5483.‏ (1965).

[42] Ibrahim, H. *et al.* Evaluation and prediction of groundwater quality for irrigation using integrated water quality indices, machine learning models and GIS approaches A representative case study. *Water* **15**(4), 694 (2023).

[43] Alezabawy, A. K. *et al.* Characterization and Evaluation of Groundwater in Wadi Abu Gurdi, West Baranis area, Southeastern Desert Egypt: Geophysical and hydrogeochemical investigations. *Front. Sci. Res. Technol.*10.21608/fsrt.2022.152965.1067 (2022). 10.21608/fsrt.2022.152965.1067

[44] Alezabawy, A. K. *et al.* Delineation and evaluation of the groundwater of fractured limestone Aquifer at East of Al Kurimat Area, Egypt: Geophysical and hydrogeochemical constraints. *Pure Appl. Geophys.* **178**(11), 4425–4459 (2021).

[45] Gad, M. *et al.* Groundwater quality and health risk assessment using indexing approaches, multivariate statistical analysis, artificial neural networks, and GIS techniques in El Kharga Oasis, Egypt. *Water* **15**(6), 1216 (2023).

[46] Nasher, N. R. & Ahmed, M. H. Groundwater geochemistry and hydrogeochemical processes in the Lower Ganges-Brahmaputra-Meghna River Basin areas, Bangladesh. *J. Asian Earth Sci. X* **6**, 100062 (2021).

[47] Glover, E. T. *et al.* Major ion chemistry and identification of geochemical processes of groundwater in the Accra Plains. *Geoscience* **50**, 10279–10288 (2012).

[48] Vasu, D. *et al.* Influence of geochemical processes on hydrochemistry and irrigation suitability of groundwater in part of semi-arid Deccan Plateau, India. *Appl. Water Sci.* **7**, 3803–3815 (2017).

[49] Drever, J. I. *The geochemistry of natural waters: surface and groundwater environments* 436 (Prentice-Hall, 1997).

[50] Datta, P. S. & Tyagi, S. K. Major ion chemistry of groundwater in Delhi area: chemical weathering processes and groundwater flow regime. *J. Geol. Soc. India* **47**, 179–188 (1996).

[51] Zaghlool, E. Geochemical modeling and statistical analysis for groundwater evolution assessment in Wadi Qasab, Sohag, Eastern Desert, Egypt. *J. Geosci. Environ. Prot.* **8**(09), 33 (2020).

[52] Clark, I. D. & Lauriol, B. Aufeis of the Firth River basin, northern Yukon, Canada: Insights into permafrost hydrogeology and karst. *Arct. Alp. Res.* **29**(2), 240–252 (1997).

[53] Craig, H. The standard for reporting concentrations of deuterium and oxygen-18 in natural waters. *Science* **133**(3467), 1833–1834 (1961). 17819002 10.1126/science.133.3467.1833

[54] Salem, Z. E. *et al.* Origin and characteristics of brackish groundwater in Abu Madi coastal area, Northern Nile Delta, Egypt. *Estuar. Coast. Shelf Sci.* **178**, 21–35 (2016).

[55] Hussein, E. *et al.* Groundwater quality assessment and irrigation water quality index prediction using machine learning algorithms. *Water* **16**(2), 264 (2024).

[56] Wilcox, L. V. Classification and Use of Irrigation Waters. US Department of Agriculture. p. 969 (1955).

[57] Richards, L. A. Diagnosis and improvement of saline alkali soils. *J. Soil Sci.* **78**(2), 154 (1954).

[58] Doneen, L.D. Salinity criteria of agricultural waters for peaty and sedimentary soils of the lower delta. University of California.(1962).

[59] Golden Software, LLC, Surfer^®^ 16.6.484 (64-bit) (2019). www.goldensoftware.com

[60] QGIS 3.38.1 (2024). https://qgis.org/

[61] Abdel Daiem AA. Melioration hydrogeology in west El-Nubaria area. PhD Thesis, Mansoura University, Egypt (1976).

[62] Conoco, Geologic map of Egypt (20 sheets, Scale 1:500,000). Egyptian General Authority for Petroleum (UNESCO Joint Map Project), Cairo. (1987).

[63] Microsoft Corporation. (2018). Microsoft Excel. https://office.microsoft.com/excel

